# Reproducibility of qualitative assessment of stent struts coverage by optical coherence tomography

**DOI:** 10.1007/s10554-012-0030-8

**Published:** 2012-03-14

**Authors:** Salvatore Brugaletta, Hector M. Garcia-Garcia, Josep Gomez-Lara, Maria D. Radu, Ravindra Pawar, Jamal Khachabi, Nico Bruining, Manel Sabaté, Patrick W. Serruys

**Affiliations:** 1 Z120, Thoraxcentre, Erasmus MC, ‘s-Gravendijkwal 230, 3015 CE Rotterdam, The Netherlands; 2 Department of Cardiology, Thorax Institute, Hospital Clinic, Barcelona, Spain; 3 Cardialysis BV, Rotterdam, The Netherlands

**Keywords:** OCT, Coverage, Strut, Stent

## Abstract

Assessment of stent strut coverage by optical coherence tomography (OCT) is not standardized. The methodology most commonly used is based on a visual binary qualitative assessment (strut covered or not). However, the influence of magnification (zoom setting) to the inter- and intra-observer agreements has not yet been evaluated. Aim of our study was therefore to evaluate the agreements of this approach, taking into account various zoom settings. 126 struts from 10 selected frames were independently evaluated by four observers using a stepwise approach increasing the zoom setting as following: (1) full view of the lumen (FV), (2) half view of the lumen (HV) and (3) a quarter view of the lumen (QV). Intra- and inter-observer agreements (κ) were assessed. The rate of uncoverage was determined for each strut as the number of times it was defined as uncovered divided by the total number of observations (maximum 12 = 3 zoom settings × 4 analysts) and expressed as percentage. The inter-observer κ values (mean [range]) were 0.32 [0.07–0.63], 0.40 [0.18–0.69] and 0.33 [0.09–0.6], within FV, HV and QV respectively. The intra-observer κ values were 0.60 [0.50–0.70], 0.75 [0.75–0.76] and 0.60 [0.50–0.70], within FV, HV and QV respectively. By increasing zoom setting the κ value of intra-observer agreement was 0.74 [0.58–0.83] (from FV to HV), 0.70 [0.56–0.83] (from HV to QV) and 0.70 [0.37–0.86] (from FV to QV). Overall, the rate of uncoverage was 15.5% [8.3–100%]. The OCT qualitative evaluation of strut coverage has wide inter and intra-observer agreements and is dependent of the zoom setting used during the analysis. A more reproducible approach would be needed to eventually increase the probability to link uncovered struts with clinical events.

## Introduction

Optical coherence tomography (OCT) is a light-based imaging modality that can provide in vivo high-resolution images of coronary stents, with detailed information about struts apposition and tissue coverage [1]. Pathological studies have suggested that the absence of stent strut coverage due to delayed vascular healing and the persistence of fibrin may be the most important determinants of late stent thrombosis, together with lesion and procedure-related settings [2, 3]. For this reason, coverage at strut-level analysis by OCT is the most common surrogate endpoint in OCT studies, providing a measurable variable for the comparison between different stents and being also an important parameter for the approval of new drug eluting stents by regulatory agencies [4].

The inter- and intra-observer reproducibility for strut count, strut apposition and quantitative strut tissue coverage measurement (e.g. neointima thickness) has been shown to be good [5, 6]. However, strut coverage evaluation is not standardized. The most commonly used approach is the visual qualitative assessment, evaluating strut coverage as a binary variable (covered or not covered). Moreover, independently from the methodology used, the zoom setting used for magnifying the OCT images may influence the assessment and thereby the reproducibility of strut coverage assessment.

The objective of the present study was to revise this qualitative approach for the assessment of strut coverage by measuring the inter- and intra-observer agreement and evaluating the influence of various zoom settings.

## Methods

### Study population

From our OCT database all patients, who received an OCT pullback at 6 months after stent implantation, were selected. After an initial quality check, an independent analyst (not involved in the assessment of coverage) randomly selected 10 frames from the OCT pullbacks identifying 126 struts, according to the following definitions:

- Highly reflective surface with cast dorsal and radial shadows;
- Highly reflective surface without dorsal shadowing.

The stent implanted was Resolute Endeavor in all the frames analyzed. Struts were termed “covered” by OCT if tissue could be identified above the struts, as previously defined [7].

### OCT acquisition

The OCT acquisition was performed using a commercially available system for intracoronary imaging (C7XR Fourier-Domain System; LightLab Imaging, Westford, Massachusetts). Pullback was performed during continuous injection of contrast medium (3 mL/s, Iodixanol 370, Visipaque, GE Health Care, Cork, Ireland) through the guide catheter with an injection pump. The automated pullback rate was 20 mm/s and the frame rate was 100 images/s.

### Qualitative evaluation of strut coverage

**Fig. 1:**
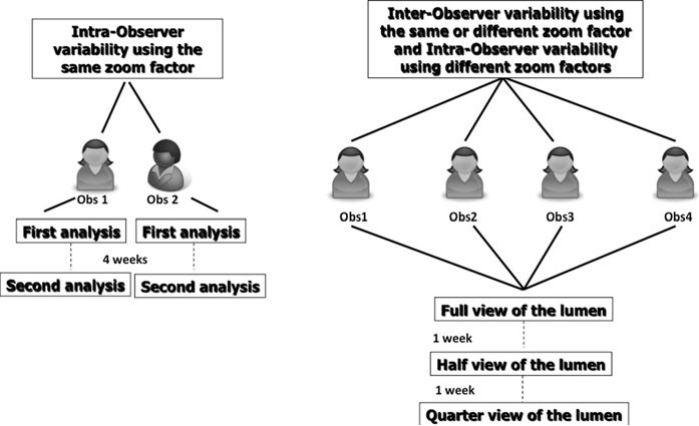
Flow-chart of the study analysis

**Fig. 2:**
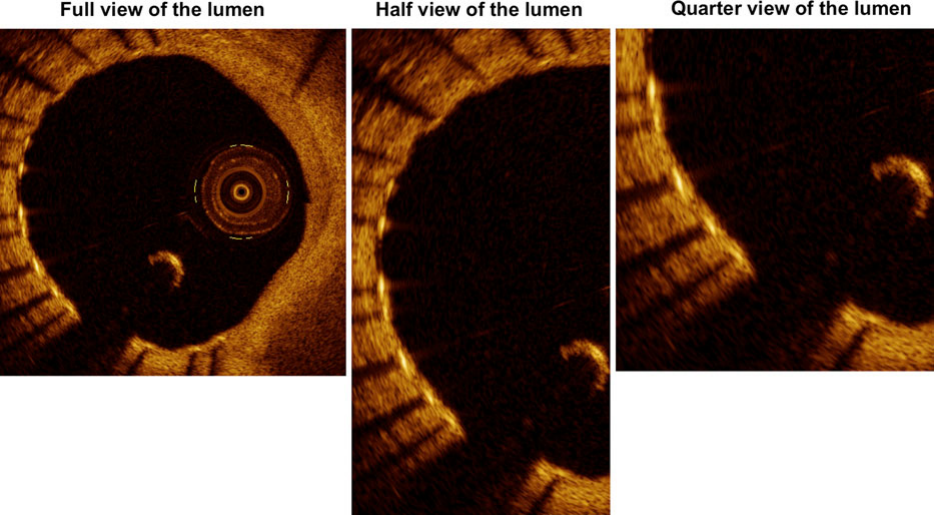
Examples of the various zoom settings used in the analysis

Four independent and expert observers separately analyzed the selected frames in order to qualify the coverage of the struts, previously defined, as a qualitative binary variable (yes/no). In particular, two analysts (Obs 1 and Obs 2) were interventional cardiologists with wide expertise in OCT evaluation; the remaining two (Obs 3 and Obs 4) were senior OCT CoreLab analysts, without experience in cardiology practice. All of them repeated the analysis with three different zoom settings 1 week later at each step in order to estimate the intra-observer agreement, related to the zoom setting (Fig. 1). Two of the analysts repeated all the analyses 4 weeks later with the same stepwise protocol in order to estimate also the intra-observer agreement within the same zoom setting. The predefined zoom settings used were: full view of the lumen, half view of the lumen and quarter view of the lumen (Fig. 2). Each strut was then evaluated 12 times (four different observers with three different zoom settings).

For the purpose of the study, the rate of uncoverage of each strut was determined according to the following formula:

$$100{\kern 1pt}\,\times \,\;\frac{\text{Number\, of\, times\, a\, strut\, is\, defined\, as\, uncovered}}{\text{Total\, number\, of\, observation}}\,$$

### Statistical analysis

The agreement in the number of struts evaluated as covered was estimated by the kappa test for agreement. The kappa values are presented as mean and range within the various zoom setting. According to previous publications: ≤0 indicates poor agreement, 0–0.20 indicates slight agreement, 0.21–0.40 indicates fair agreement, 0.41–0.60 indicates moderate agreement, 0.61–0.80 indicates good agreement, and 0.81–1.0 indicates excellent agreement [8, 9]. Wilcoxon paired test was used to compare the number of struts assessed as covered between the various zoom factors. Comparison between groups was performed by Mann–Whitney test. Data were analyzed with SPSS version 16.0 software (SPSS Inc., Chicago, IL).

## Results

### Inter-observer agreement within the same zoom setting

Table 1 reports the inter-observer agreements according to the various zoom settings used. The κ values were 0.32 [0.07–0.63], 0.40 [0.18–0.69] and 0.33 [0.09–0.65], from the full view of the lumen, through half view up to quarter view of the lumen, respectively. Out of 126 struts, the average number of the struts evaluated as uncovered by the analysts was 21.5 [range: 3–50] using the full view of the lumen, 20.2 [7–46] with half view of the lumen and 17.2 [3–42] with quarter view of the lumen.

**Table 1:** Inter-observer agreement within a same zoom setting

	Observer 1	Observer 2	Observer 3	Observer 4
Full view of the lumen				
Observer 1		0.40	0.07	0.20
Observer 2	0.40		0.25	0.63
Observer 3	0.07	0.25		0.41
Observer 4	0.20	0.63	0.41	
Half view of the lumen				
Observer 1		0.69	0.18	0.45
Observer 2	0.69		0.18	0.45
Observer 3	0.18	0.18		0.47
Observer 4	0.45	0.45	0.47	
Quarter view of the lumen				
Observer 1		0.65	0.09	0.25
Observer 2	0.65		0.18	0.37
Observer 3	0.09	0.18		0.47
Observer 4	0.25	0.37	0.47	

Κ value are reported

Overall, there was on average a progressive decrease in the struts detected uncovered going from full to half view of the lumen (−5.8%; *p* = 0.275), from full to quarter view of the lumen (−14%; *p* = 0.001), and from half to quarter view of the lumen (−17%; *p* = 0.018).

### Intra-observer agreement changing the zoom setting

Increasing the zoom setting from full to half view of the lumen, the intra-observer agreement (k-value) was 0.74 [0.58–0.83], while from half to quarter view of the lumen it was 0.70 [0.56–0.83] and from full to quarter view of the lumen 0.70 [0.37–0.86] (Table 2). In particular, the intra-observer agreement was higher within Obs 3 and 4 (senior OCT CoreLab analysts) than within Obs 1 and 2 (interventional cardiologists with wide expertise in OCT evaluation) (0.82 [0.76–0.86] vs. 0.61 [0.37–0.76]; *p* = 0.002).

**Table 2:** Intra-observer agreement, according to the zoom setting

From lumen to half view of the lumen	
1st Observer	0.58
2nd Observer	0.76
3rd Observer	0.83
4th Observer	0.80
From lumen to quarter view of the lumen	
1st Observer	0.65
2nd Observer	0.56
3rd Observer	0.83
4th Observer	0.76
From half to quarter view of the lumen	
1st Observer	0.37
2nd Observer	0.75
3rd Observer	0.86
4th Observer	0.85

Κ value are reported

### Intra-observer agreement within the same zoom setting

Two analysts have assessed the intra-observer agreement within the same zoom setting. Using full view of the lumen the average k-value of agreement for each observer was 0.60 (0.70 and 0.50 for the two observers, respectively), using half view of the lumen it was 0.75 (0.76 and 0.75 for the two observers, respectively) and using quarter view of the lumen 0.60 (0.50 and 0.70 for the two observers, respectively).

### Rate of uncoverage of the struts

**Fig. 3:**
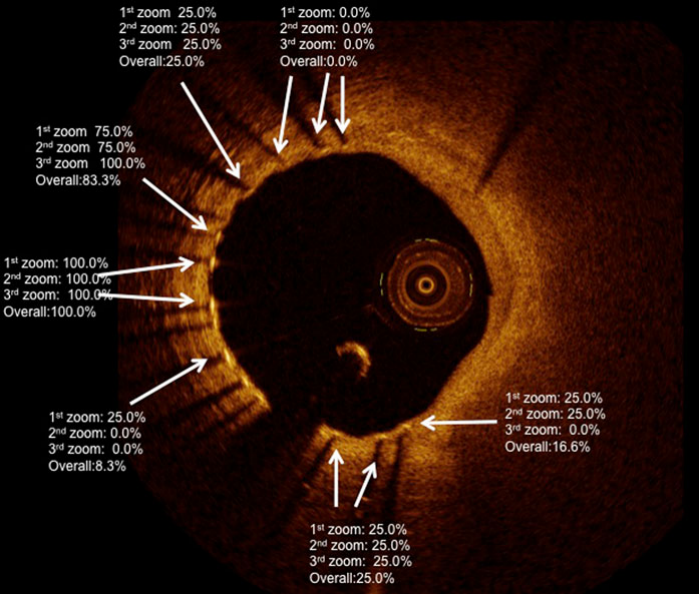
Example of the wide variability of the rate of uncoverage, according to the various zoom settings used in the analysis. Some struts are adjudicated by all the analysts as uncovered, independently from the zoom setting (rate of uncoverage 100%). Some struts have a probability to be defined as uncovered from 8.3 to 83.3%

Overall, the rate of uncoverage for each strut was 15.5% [8.3–100%]. Within full view of the lumen it was 16.9% [25–100%], half view 16.0% [25–100%], quarter view 13.6% [25–100%] (Fig. 3).

## Discussion

Our analysis demonstrates a wide inter- and intra-observer agreement for uncovered struts evaluated visually by OCT, which is highly dependent of the zoom setting used in the analysis.

Vascular healing of metallic stent has been extensively studied by anatomo-pathologists, as the delaying of this process has been related to the occurrence of very late stent thrombosis [2]. In vivo, OCT is highly suited for the evaluation of strut coverage due to its high resolution and image quality [10]. Assessment of stent strut coverage, however, is not standardized and a quantitative or qualitative approach can be used. In the first methodology, struts coverage is evaluated through quantification of tissue coverage area: the operator manually traced the stent and lumen area, deriving the tissue coverage area. Using this kind of approach, a good intra- and inter-observer agreement has been reported for the neointima thickness measurement [5, 11, 12]. In a further step, in order to report these data at strut level, a predefined threshold of neo-intima thickness is used in a semi-quantitative fashion to define a strut as covered [13, 14]. However, some important concerns must be highlighted in the interpretation of these results. The use of a threshold is quite arbitrary and has some therein limitations, as it is not standardized for different stent and even for the same stent. In addition, Murata et al. comparing the morphometric differences at the strut level between OCT and histology have shown that the correlation between these techniques is much dependent on the amount of the neointima present. OCT seems to correlate appropriately with histology only in either the absence (<20 μm) or the presence (>100 μm) of robust neointima. It should be also considered that the strut blooming is about 37 μm in thickness and extends bi-directionally toward and away from the catheter light source, complicating the measurement of low neointimal coverage (<20 μm) [15]. Of note is that 10–20 μm represents the OCT resolution and that the majority of the drug eluting stents report a neointima thickness between 20 and 100 μm. For these reasons, the choice of an arbitrary threshold should be carefully considered. In addition, the zoom setting used in the analysis, seldom reported in the majority of the OCT-stent papers, is not standardized and can further increase the variability of the assessment.

The second and most used approach to evaluate the strut coverage is a visual qualitative classification of strut coverage as a binary variable (covered or not covered) [16]. This qualitative assessment is sometimes performed at a distance interval different from that used in the quantitative OCT measurement (e.g. each frame vs. 0.33 mm interval) and no reproducibility is reported [17]. The zoom setting, of utmost importance in this qualitative evaluation as compared to the quantitative approach, is neither standardized nor specified. In our analysis, we tested the agreement of this approach in a Core Lab, using different zoom settings. We demonstrated that the zoom setting is an important bias and the range of intra-observer agreement according to the zoom used is very wide: a same strut can range from 0 to 25% probability to be considered as uncovered, using different zoom. In particular, moving from the first (lumen) to the second zoom (half lumen), there was a slight decrease in the number of the strut detected as uncovered. Increasing further the zoom (quarter lumen), there was a significant decrease in the struts detected as uncovered. Using the same zoom setting, the intra-observer agreement was on average good, but with wide variability. Although overall the inter and intra-agreement was not high (e.g. close to 1), looking at the various k-value per zoom factor the half view of the lumen appeared as the zoom factor with a higher reproducibility between and within the observers as compared to the others and could be used as a reference in future studies.

Of note is that in a careful evaluation of our results two different kind of analysts can be identified: the Core Lab analysts (Obs 3 and Obs 4) showed a good intra-observer agreement changing the zoom setting, compared to the interventional cardiologists with wide experience in OCT evaluation (Obs 1 and Obs 2), who exhibited a low agreement in their measurements changing the zoom setting. This observation supports the presence of a CoreLab for performing such analysis, which should be blinded to the clinical meaning of the measurements and only based on a phenomenological description of strut coverage.

The poor agreement in qualitative assessment of strut coverage raises some concerns about the clinical interpretation of these findings. OCT was, indeed, advocated as the gold standard to evaluate the reliability of the degree of incomplete coverage, identifying those patients at increased risk of late stent thrombosis. Nevertheless, it is unable to distinguish between fibrin, giant cells, granulomatous reaction and degree of endothelization and our results could be considered as supportive of this limitation, as each analysts thinks differently about the “status” of coverage of each strut.

### Limitations

A small number of frames have been analyzed in the current study to allow multiple evaluations by the 4 different observers. The fact that the k-value could improve by increasing the number of observation should be acknowledged. Nevertheless, it is of note that four different observers, who assessed the frames up to 4 times, have been used in our analysis.

## Conclusions

Qualitative evaluation of strut coverage by OCT has wide inter and intra-observer agreements, extremely dependent from the zoom setting used during the analysis. 
